# Long COVID-19, persistent somatic symptoms and social stigmatisation

**DOI:** 10.1136/jech-2021-216643

**Published:** 2021-02-23

**Authors:** Aranka Ballering, Tim Olde Hartman, Judith Rosmalen

**Affiliations:** 1 University Medical Centre Groningen, Interdisciplinary Center for Psychopathology and Emotion Regulation, University of Groningen, Groningen, The Netherlands; 2 Department of Primary and Community Care, Radboudumc, Nijmegen, The Netherlands

**Keywords:** COVID-19, communicable diseases, psychosocial factors

We applaud the efforts of van Daalen *et al* to raise awareness about social stigma towards COVID-19.^1^ The authors provide multiple examples of how social stigma is harmful for COVID-19 containment on a global scale, as well as on an individual level. The infectious nature of COVID-19 ensures that social stigmatisation of patients is rooted in a fear of contagiousness, accompanied by beliefs that patients are to blame, and thus responsible, for their disease. These feelings of blame towards patients often perpetuate after recovery.^1^ The phenomenon of perpetuated blame is especially salient given the longevity of COVID-19 symptoms in some patients after their infection has cleared: long COVID-19. Although the definition of long COVID-19 remains debatable, it is generally accepted that long COVID-19 is defined by persistent symptoms that are still reported 3 weeks post-infection.^2^ People that have recovered from COVID-19, but are still experiencing symptoms are no longer contagious. However, they still can be stigmatised by bystanders and healthcare professionals.

Long COVID-19 bears a resemblance to functional somatic syndromes characterised by persistent somatic symptoms of unclear aetiology. Such syndromes often develop after an eliciting trigger, such as a viral infection. However, at the moment of symptom reporting, no clear somatic abnormalities can be found despite sound history taking and diagnostic investigation. The absence of detectable bodily abnormalities in people affected by persistent somatic symptoms facilitates stigmatisation. This stems from dualistic thinking, that is, the body-versus-the-mind idea, which allows others, including healthcare professionals, to assume patients should ‘toughen up’ as apparently nothing is physically wrong.^3^ Thus, in persistent somatic symptoms social stigmatisation stems from the psychosomatic connotation of symptoms: the blame projected towards people affected by persistent somatic symptoms refers to the perceived inability of people to waver their symptoms.^4^ These negative attitudes are likely to negatively impact help-seeking behaviour for these symptoms as is commonly seen in other (infectious) diseases.^5^

**Figure 1:**
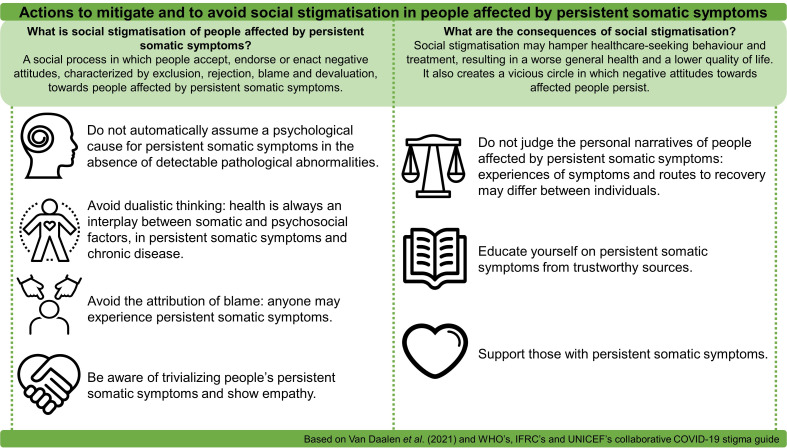
Actions to mitigate and to avoid social stigmatisation in people affected by persistent somatic symptoms.

We agree with van Daalen *et al* that social stigmatisation is a risk factor in COVID-19 mitigation. However, we should not overlook social stigmatisation of people affected by persistent somatic symptoms by bystanders and healthcare professionals. Stigmatisation should be avoided, as it negatively influences health-seeking behaviour and quality of life of those affected. Figure 1 describes potential steps on an individual level that ameliorate social stigmatisation towards people affected by persistent somatic symptoms, which is particularly urgent given the predicted increase of people affected by long COVID-19.
